# Minimizing the knowledge-to-action gap; identification of interventions to change nurses' behavior regarding fall prevention, a mixed method study

**DOI:** 10.1186/s12912-021-00598-z

**Published:** 2021-05-21

**Authors:** Lysette Hakvoort, Jeroen Dikken, Maaike van der Wel, Christel Derks, Marieke Schuurmans

**Affiliations:** 1 Mxima MC, De Run 4600, 5504 DB Veldhoven, The Netherlands; 2 De Haagse Hogeschool, Faculteit Gezondheid, Voeding & Sport, Johanna Westerdijkplein 75, 2521 EN The Hague, The Netherlands; 3 Sint Franciscus Gasthuis en Vlietland, Kleiweg 500, 3004 BA Rotterdam, The Netherlands; 4 Elisabeth Tweesteden Ziekenhuis, Dr. Deelenlaan 5, 5042 AD Tilburg, The Netherlands; 5 Onderwijscentrum, UMC Utrecht, Heijmans van den Berghgebouw, 3508 GA Utrecht, The Netherlands

**Keywords:** Falls, Aged, Behavior, Continuing nursing development, Continuing nursing education, Hospitals, Nurses, Primary prevention

## Abstract

**Background:**

The need for effective continuing education is especially high in in-hospital geriatric care, as older patients have a higher risk of complications, such as falls. It is important that nurses are able to prevent them. However, it remains unknown which interventions change the behavior of nurses. Therefore, the aim of this study is to identify intervention options to change the behavior of hospital nurses regarding fall prevention among older hospitalized patients.

**Methods:**

This study used a mixed method design. The Behavior Change Wheel (BCW) was used to identify intervention functions and policy categories to change the behavior of nurses regarding fall prevention. This study followed the eight steps of the BCW and two methods of data collection were used: five focus groups and three Delphi rounds. The focus groups were held with hospital nurses (*n*=26). Geriatric experts (*n*=11), managers (*n*=13) and educators (*n*=13) were included in the Delphi rounds. All data were collected within ten tertiary teaching hospitals in the Netherlands. All participants were included based on predefined in- and exclusion criteria and availability.

**Results:**

In Geriatric experts opinions interventions targeting behavior change of nurses regarding fall prevention should aim at after-care, estimating fall risk and providing information. However, in nurses opinions it should target; providing information, fall prevention and multifactorial fall risk assessment. Nurses experience a diversity of limitations relating to capability, opportunity and motivation to prevent fall incidents among older patients. Based on these limitations educational experts identified three intervention functions: Incentivisation, modelling and enablement. Managers selected the following policy categories; communication/marketing, regulation and environmental/social planning.

**Conclusions:**

The results of this study show there is a discrepancy in opinions of nurses, geriatric experts, managers and educators. Further insight in the role and collaboration of managers, educators and nurses is necessary for the development of education programs strengthening change at the workplace that enable excellence in nursing practice.

**Supplementary Information:**

The online version contains supplementary material available at 10.1186/s12912-021-00598-z.

## Introduction

Healthcare systems require continuous innovation to meet the needs of patients and providers. Innovation and changes in healthcare, ask for nurses to continuously improve their knowledge and skills to provide safe, efficient and effective care [1]. In other words, current developments in healthcare systems require continues change in behavior of nurses which is mainly addressed by continuing education [2]. Furthermore, there is a growing awareness that research findings are not making their way into practice. This stimulates increased interest in finding ways to minimize what is described as the knowledge-to-action gap. It requires a transfer of knowledge in which nurses are able to apply new knowledge into practice (i.e. change their behavior) [3]. Despite a growing body of empirical research in this topic, the effectiveness and impact of continuing education in clinical practice remains underexplored. Does education really change the behavior of professionals?

There are several reasons why continuous education in nursing practice needs attention. First, continuous innovation demands that nurses develop dispositions that are crucial to transfer new knowledge into practice [2], i.e. change their behavior, such as creative and critical thinking, reflection skills and research awareness. Professional education tended to focus on acquisition of new knowledge (knows how) instead its application into practice (does). Continuing education remains largely didactic, even though the importance of shifting it to more participatory education has been noted in research. Second, scarcity of follow-up evaluations of continuing educational programs lead to a lack of evidence on how education can improve patient outcomes or how theoretical knowledge is applied in practice [4]. Third, nurses experience several inhibitors for participating in education programs. Often the education programs are mandatory, which results in a lack of intrinsic motivation [5]. Furthermore, there are challenging conditions such as a high number of participants, shortage of budget, planning of educational activities and lack of familiarity with nurses educational needs [4, 6]. As nurses with state-of-the-art knowledge and skills are conditional for quality of care, the need for more evidence relating to the effect of educational programs is necessary [4].

This is especially relevant for the geriatric care. Worldwide, people are aging [7]. Meaning that hospital nurses increasingly encounter older patients in their daily work. Hospitals are a potential dangerous place for these patients, because of a higher risk for complications, such as falls [8,10]. International incidence numbers of in-hospital falls vary between 2 and 15% and costs resulting from falls alone have been reported between 0.851.5% of the total healthcare expenses within the United States, Australia, European Union and United Kingdom [11, 12]. Moreover, older people who experience falls report increased anxiety and a reduced quality of life [13]. Because of the serious consequences of in-hospital falls, such as injuries, functional decline and prolonged hospital stays [10, 14], it is important that nurses have the right attitudes, knowledge and skills to prevent them. Especially, because the possible lack of a caring attitude and knowledge among nurses is suggested as a barrier for successful fall prevention [15].

The transfer of new knowledge into practice requires behavior change. It is however unknown which intervention functions and policy categories target behavior change, in this study the behavior of nurses regarding fall prevention. Therefore, the aim of this study is to identify intervention options to change the behavior of hospital nurses regarding fall prevention among older hospitalized patients. Results of this study can then be used in the development of an education program regarding fall prevention.

## Methods

### Design

This study used a mixed method. The eight steps of the Behavior Change Wheel were followed (Table1) in which two methods of data collection were used: five focus groups and three Delphi rounds [16, 17] Data was collected between December 2018 and July 2019. All methods were carried out in accordance with relevant guidelines and regulations. Written informed consent was obtained from all study participants. This study was approved by the ethics medical board of MMC (Maxima Medical Center) in Brabant, the Netherlands (N.18.146).

**Table 1:** Stages, steps, method of data collection and respondents^a^

Stages of BCW	Steps of BCW	Method	Respondents
**1; Understand the behavior**	1; Define the problem in behavioral terms	Delphi, method of Lynn	Experts in Geriatrics
			*First round, n=11*
			*Second round, n=10*
	2; Select target behavior		
	3; Specify the target behavior		
	4; Identify what needs to change	Focus groups, thematic analyses	Hospital nurses *n=26*
**2; Identify intervention options**	5; Intervention functions	Delphi, thematic analyses	Educational experts *n=13*
	6; Policy categories		Managers *n=13*
**3; Identify content and implementation options**	7; Behavior change techniques	Delphi, thematic analyses	Educational experts *n=10*
	8; Mode of delivery		

^a^The first two columns show the stages and steps of the BCW. Per step the used method for data collection is explained and the number (n=) and type of respondents that were included

### Behavior change wheel

To minimize the knowledge-to-action gap, a framework targeting behavior change was used. There are several frameworks developed to address the complexity in defining, developing and evaluating interventions to change behavior, such as the Behavior Change Wheel (BCW). The BCW was developed from 19 frameworks of behavior change andconsists of three stages and eight steps to change a target behavior (Table 1), in this study the behavior of nurses regarding fall prevention. At the center of the BCW lies the COM-B model (Capability, Opportunity, Motivation-Behavior). In the COM-B model behavior is a result of an interaction between three components, which includes: Capability, which can be psychological (knowledge) or physical (skills); Opportunity can be social (societal influences) or physical (environment); motivation can be automatic (emotion) or reflective (beliefs, intentions) [16, 17].

### Data collection

In stage one (understand the behavior), we used a two round Delphi, using the method of Lynn, with experts in geriatrics (steps one to three) and focus groups, using thematic analyses, with hospital nurses (step four).

In stage two (identify intervention options) and three (identify content and implementation options) we used again a Delphi round, using thematic analyses, with educational experts (step five, seven and eight) and a Delphi round with managers (step six) (Table 1).

### Stage one; understand the behavior (steps one to four)

A two-round Delphi, using opinions of experts in geriatrics, was used to define, select and specify the target behavior (steps one to three). Eligible experts were medical doctors, nurse practitioners, nurse specialists and physiotherapists with further education in geriatrics. All experts worked in one of the ten tertiary Dutch teaching hospitals affiliated with the Research, Education and Nursing regarding Elderly (RENursE) consortium. Stage one of the BCW includes a lot of data and information on which consensus among experts is needed. Therefore, the method of Lynn was used to help with this process. Lynn is a quantification of a qualitative process, meaning that the item scores are interpretative and meaning or conclusions are based on context and interpretation [18]. Experts answered open and closed questions. Closed questions were scored on a four-point Likert scale: *irrelevant, mainly not-relevant, mainly relevant, and highly relevant*. For closed questions an item-content validity index (I-CVI) was determined. The I-CVI refers to the number of experts who defined the content relevant. The method of Lynn requires a minimum of five members as suggested in literature [19]. Therefore we aimed to include one or two experts per hospital for each Delphi round.

In the first Delphi round experts were presented a definition of the behavior of nurses to prevent falls based on the Dutch guideline for fall prevention among older hospitalized patients [20]. Experts were asked to determine the relevance and completeness of the definition. Next, experts were asked to determine which behaviors were most relevant for changing the behavior of nurses from a list of seven behaviors described in the Dutch guideline for fall prevention [20]. Relevance was determined by answering four questions per behavior, relating to impact on the outcome, ease of change and measurement and impact on other related behaviors (Additionalfile2) [16, 17]. As a result of the first round a top three of target behaviors was derived, based on the I-CVI scores. In the second round, experts were provided an overview of the seven behaviors including the scores of the first round and were asked to give feedback and confirm the relevance of the top three of target behaviors.

For step four, semi-structured focus groups with nurses were used to identify their current behavior and perspectives regarding fall prevention. Eligible nurses needed to work at a ward where older patients were admitted, in one of the RENursE hospitals. Nursing students were excluded because of possible limited experience in caring for older adults. The aim was to reach a-priori thematic saturation based on the COM-B and to include six to eight nurses per focus group to ensure a variety of perspectives, but also small enough to prevent disorder [16, 17] Nurses were asked about the themes: capability (knowledge and skills), opportunity (social and environmental influences) and motivation (emotional and reflective) regarding fall prevention. Furthermore, a patient case was presented during the focus groups to identify the current behavior of nurses. Based on the patient case nurses shared step-by-step the interventions they used (Additional file 1). Finally, nurses were asked which behaviors of the Dutch guideline of fall prevention should be included in a fall prevention program. This way, the opinions of geriatric experts and nurses could be compared.

### Stage two; identify intervention options (steps five and six)

A Delphi round with educational experts was used to identify intervention options (step five). Educational experts were included for the innovative and didactic choices. All educational experts worked in one of the RENursE hospitals. Educational experts received the results of the focus groups (step four) with the opinion of nurses described per COM-B factor. In the first Delphi round they were asked to complete a table in which intervention options could be selected per COM-B factor. This table is provided in the BCW. As it was important to understand the choices the experts made in their intervention options, we used open questions which were analyzed using thematic analyses.

A one round Delphi was used to include the opinions of managers about policy categories to support or change the behavior of nurses (step six). Experts in management were middle managers as they are positioned between the wards and higher management with first-line responsibilities [21] All managers worked in one of the RENursE hospitals. Managers received the results of the focus groups with nurses described per COM-B factor. They were also asked to complete a table in which policy categories could be selected per COM-B factor. This table was provided in the BCW. Qualitative feedback about the reason for their choices and how they would use or implement the policy categories was also asked in this Delphi round and analyzed with thematic analyses.

### Stage three; identify content and implementation options (steps seven and eight)

In the second Delphi round with the educational experts (after step five), the most selected intervention options per COM-B factor were shared with the educational experts and qualitative feedback was asked about how the intervention options could change the behavior of nurses and what modes of delivery were possible (step seven and eight).

### Setting

This study was embedded in ten tertiary teaching hospitals in the Netherlands, affiliated in the RENursE consortium. All participants included in this study worked in one of the RENursE hospitals and were selected based on in- and exclusion criteria and availability.

### Procedures

#### Delphi rounds with experts in stages one to three of the BCW (steps one to three and five to eight)

**Fig. 1:**
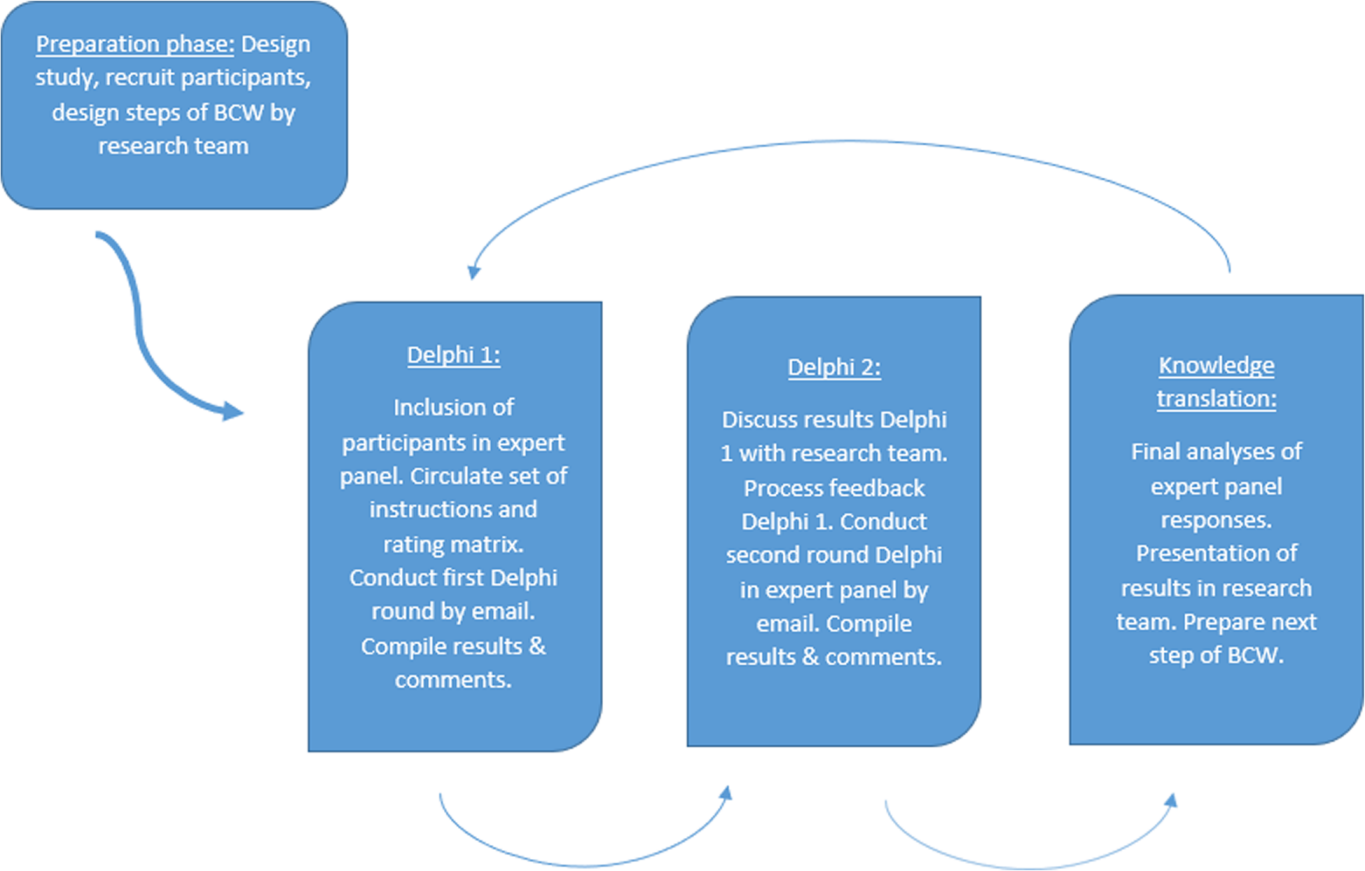
Step-by-step process of the Delphi rounds. In the first step the participants were included in the expert panel and they received the first instructions and rating matrix. In the second round the results were discussed within the research team and additional questions for the expert panel were formulated. The expert panel received the additional questions. In the last step, knowledge translation, the research team analyzed and discussed the final results

Each RENursE hospital has a nurse researcher (NR). The NR approached one or two experts from their own hospital by email including the information letter of the study. This way, 10 to 20 experts were included in each Delphi. Included experts gave written consent and were contacted by the main researcher with further information about the study procedures and instructions. Further contact was by email, including sending and receiving questionnaires in the Delphi rounds. After each round data was processed and discussed within the research team and the next step of the BCW was prepared (Fig.1).

#### Focus groups with hospital nurses in stage one of the BCW (step four)

All NRs of RENursE were asked to organize a focus group in their own hospital including six to eight nurses to ensure a variety of perspectives. All nurses gave written consent. The main and second researcher travelled to the hospitals for the focus groups. The main researcher was the interviewer, the second researcher made field notes, drew diagrams with the names of participants and helped with practical matters. All focus groups were audio-recorded and transcribed.

### Analyses

#### Method of Lynn

For closed questions in the Delphi an item-content validity index (I-CVI) was determined. The I-CVI refers to the number of experts who defined the content relevant. All scores of the experts were dichotomized in to relevant (mainly relevant/ highly relevant=1) and not relevant (irrelevant/ mainly not-relevant=0). Than the I-CVI was calculated: sum of the scores of the item / number of experts. An I-CVI score0.78 was considered relevant [18].

#### Thematic analyses

A thematic analysis according to Braun and Clarke consisting of six phases was used to analyze the data of the focus groups [22, 23]. First the main and second researcher familiarized themselves with the data by transcribing all the focus group material and reading the transcribed material. The main and second researcher analyzed the transcripts independently. The initial codes formed a list of ideas about the data (open codes) and were then organized in broader categories based on repeated patterns (axial codes). The COM-B was kept in mind, but the open and axial codes were primarily data driven. Then the open and axial codes were combined with the pre-defined components of the COM-B (Capability, Opportunity, Motivation-Behavior).

### Trustworthiness

Trustworthiness was achieved by several strategies. Data triangulation was used by including multiple data sources; nurses, geriatric and educational experts and managers which increased the validity. Nurses, experts and managers were included from ten different hospitals in different areas of the Netherlands, resulting in a maximum heterogenic sample. Investigator triangulation increased the credibility of the data. A second researcher independently analyzed the data of the focus groups and results were discussed within the research team to foster reflexivity. This way any biases of the main researcher were discussed and reflected on. Theory and methodological triangulation was reached by including method of Lynn and COM-B as theories. Also, during the focus groups the five stages for focus group researchers were used to establish ground rules and consider ethical issues [24]. At last, the consolidated criteria for reporting qualitative research (COREQ) were used to write this article [25].

## Results

### Stage one; understand the behavior (steps one to four)

The Delphi included 13 geriatric experts from six hospitals. Data was collected from December 2018-Februari 2019. Experts consisted of nurses (*n*=5), physiotherapist (*n*=1), medical doctors (*n*=4) and nurse specialists (*n*=3). The first round was completed by 11 experts, the second round by 10 experts. Due to work or private related issues two medical doctors did not complete both rounds and one medical doctor did not complete the second round.

All experts shared the opinion that the definition according to national guidelines was relevant (ICVI=1). Qualitative feedback of the experts resulted in two minor adjustments, resulting in the following definition: *Improvement of applying fall prevention and repressive interventions by nurses, working in hospitals, according to the national guideline; prevention of falls regarding elderly people.*

Seven target behaviors were derived from the national guideline for fall prevention [20]. Experts find after-care (care post fall), estimating fall risk and providing information the most promising target behaviors (Table2, respectively a mean I-CVI of 0.82, 0.80 and 0.79). They estimate the impact as highly relevant (respectively an I-CVI of 1.0, 0.91 and 0.91) to the desired outcome and think that the target behaviors have a relevant impact on other related behaviors (I-CVI of 0.820.73-0.82). In experts opinions estimating fall risk is a relevant target behavior as fall prevention starts with a thorough assessment, carried out by nurses when patients are admitted in a hospital. Providing the right knowledge to patients and their families is essential in preventing falls. Nurses should be able to provide this information and increase awareness among patients and families. After-care is important according to experts as hospitals should have an open registration and dialogue culture to stimulate learning opportunities. But is also important for patients as they may experience increased anxiety or other negative outcomes.

*Expert (physiotherapist): When a fall has occurred, the right after care needs to take place to prevent anxiety for falls, functional decline and prolonged hospital stay.*

**Table 2:** Relevance of seven target behaviors derived from national guideline of fall prevention

	How much impact will changing the behavior have on the desired outcome?	How likely is it that the behavior can be changed?	How likely is it that the behavior will have an impact on other, related behaviors?	How easy is it to measure the behavior?	Total ICVI score (*m*)^a^
Estimating fall risk	0.91	0.82	0.73	0.73	0.80
Risk assessment	0.82	0.82	0.64	0.45	0.68
Fall prevention	0.82	0.82	0.73	0.50	0.72
Compliance of elderly patients	0.45	0.36	0.64	0.18	0.41
Organization of care	0.82	0.82	0.73	0.36	0.68
Providing information	0.91	0.91	0.82	0.5	0.79
After-care (care post fall)	1.0	0.64	0.82	0.82	0.82

ICVI scores based on four-point Likert scale. Mainly relevant or highly relevant=1, irrelevant or mainly not relevant=0

^a^ Total ICVI score based on ICVI scores of four questions per target behavior. (m)=mean

Nurses behavior and perspectives regarding fall prevention were included by focus groups in five different RENursE hospitals. In total 26 nurses were included. Two focus groups included less than six nurses, because of last minute cancellation of the participant. The other five hospitals did not succeed in organizing a focus group due to organizational limitations. All focus groups were held in January 2019 and lasted between 55 and 75min. The average years of working experience was 12 (2-30) years. Nurses worked on different wards; surgeon ward (*n*=7), internal medicine ward (*n*=15), combination ward (n=1) and acute care ward (*n*=3).

A priori thematic saturation was reached as no new codes emerged from the data. No codes derived from the data that could not be combined with the pre-defined themes of the COM-B. The results are described per element of the COM-B. Diagrams were made for capability, opportunity and motivation elements (Additionalfiles3, 4, 5). Themes are supported by quotes with number of focus group (F), number of participant (R) and years of working experience (Y).

#### Capability (knowledge, skills)

Nurses report limited knowledge regarding fall prevention among younger colleagues, but also among medical doctors, patients and families. In nurses perspectives limited knowledge is a barrier for applying fall prevention interventions according to national guidelines.

*F4R1Y11: It helps when there is an adequate nursing team. Younger nurses, who finished their education two months ago, have limited expertise. ..Patients and family think falls are related to getting older and is considered normal.*

Although nurses report limited knowledge of others, they themselves also have unconsciously a lack of knowledge. They have limited knowledge about the presence of local and national guidelines regarding fall prevention. Several nurses did not know whether there is patient information material available in their hospital.

*F3R3Y2: .If there is a local guideline, I think so. I have to say very honestly that I do not know it, we do not use it. .information leaflets about fall prevention? I do not know. F3R3Y23: No, me neither.*

Moreover, nurses think that falls among older patients who are confused is not preventable.

*F2R3Y9: When we evaluate fall incidents, most of the time we conclude that we could have prevented it, not always though, because there are also confused older people. You can apply interventions and they still fall out of bed.*

Finally, according to nurses fall prevention is of low priority in most hospitals and does not play an important role in daily nursing practice.

*F2R3Y9: It really is underexposed. The need is not recognized. There is insufficient awareness of the need that we should do a risk assessment at admission and apply preventive interventions.*

#### Opportunity (social, environmental influences)

Nurses think that hospitals are not prepared for the increase of older people. They describe a lack of mobilization tools, there are slippery bathroom floors, wards with no toilet rails, or wrong placed grab bars and support rails.

*F2R5Y11: Toilets are not high enough, toilets are too small for save mobilization, there are no support rails. Things like that.*

Processes are often limited to single wards instead of hospital wide and therefore conditions are different. Technology is sometimes supportive and sometimes obstructive. They describe a lack of communication tools or a diversity of electronic patients files.

*F2R4Y8: .On the Geriatric ward, we use sensors, at Psychiatric ward they have extra low beds for fall prevention, at Neurology they have fall-out fall prevention mats. I think thats strange.*

#### Motivation (emotions, beliefs)

Nurses feel motivated and responsible to prevent falls as they do not want their patient to experience a fall. When nurses take control to change conditions, their efforts lead to few results because of managerial decision processes. Which effects their motivation negatively. Furthermore, nurses experience stress because of workload and limited staff occupation. Both influence their motivation in a negative way.

*F5R3Y1: Motivation comes from two ways, you do not want your patient to get injured, and when a patients falls, it causes a lot of hassle.*

#### Behavior

Based on a patient case the current behavior of nurses was identified. Nurses experience a lack of completion of the nursing assessment when patients are admitted in the evening or at night. As a result, the estimation of the fall risk is sometimes not completed. A multifactorial fall risk assessment is, according to nurses, currently not a part of fall prevention care in their hospitals. Fall prevention interventions are most of the time related to optimizing vision and hearing abilities of patients and providing tools for mobilization. Nurses experience limited multidisciplinary collaboration and therefore a lack of multidisciplinary interventions relating to mobilization and nutrition programs. And when nurses are anxious their patient will fall, they often use physical restraint interventions. Nurses find it very difficult to involve older patients in their care. Some nurses experience patients and families as a barrier because they do not listen or follow instructions. Nurses think they do not have the right knowledge and tools to provide sufficient information to them. Furthermore, nurses experience a limitedculture of safety [26]. Some hospitals do not register and evaluate fall incidents on a regular base.

*F3R3Y23: Registration of falls depend on degree of injury. F3R1Y18: It depends on who works, whether or not it is registered.*

At last nurses were asked which of the behaviors of the Dutch guideline were most promising to change. Answers were divers, but in most nurses opinions, providing information, fall prevention and multifactorial fall risk assessment are the most promising target behaviors. Arguments for these target behaviors were broad, but mainly related to the knowledge deficits nurses experience.

#### Stage two; identify intervention options (steps five and six)

In step five, the Delphi included 14 educational experts from eight different hospitals of the RENursE consortium. One educational expert did not complete the Delphi due to limited time. Data was collected between March and April 2019. Table3 shows the number of educational experts who selected certain intervention functions which are described in het BCW per COM-B component.

**Table 3:** Selection of intervention functions^a^

	Education	Persuasion	Incentivisation	Coercion	Training	Restriction	Environmental restructuring	Modelling	Enablement
Psychological capability	11	5	8		3		2	9	5
Physical Opportunity		5	1		2		10	3	10
Social opportunity	2	6	7		5	1	5	11	3
Reflective Motivation	4	6	11		3		2	9	5
Automatic motivation	2	3	8		1		7	7	6

^a^Number of educational experts who selected intervention functions

In step six, the Delphi included 13 managers from seven different hospitals of the RENursE consortium. Managers came from two different levels, namely head of nursing ward (*n*=8) and higher business managers (*n*=4). The heads presented different nursing wards; surgical ward (*n*=1), internal medicine ward (*n*=7), combination ward (*n*=2) and learning academy (*n*=3). Data was collected between May and June 2019. Table4 shows the numberof managers who selected certain policy categories which are described in het BCW per COM-B component. In the same Delphi round, managers were asked to give qualitative feedback about their choices. Results are described below per COM-B component.

**Table 4:** Selection of policy categories^a^

	Communication / marketing	Guidelines	Fiscal measures	Regulation	Legislation	Environmental / social planning	Service provision
Psychological capability	9	10	2	10	5	4	4
Physical Opportunity	3	3	9	3	2	11	8
Social opportunity	7	5	3	6	1	10	8
Reflective Motivation	7	8	3	8	2	8	5
Automatic motivation	6	5	1	6	2	8	6

^a^Number of managers who selected policy categories

#### Capability (knowledge and skills)

To stimulate psychological capability most of the managers selected communication / marketing, guidelines and regulation as policy categories. Communication and marketing is often chosen to improve knowledge and stimulate intrinsic motivation of nurses.

*P: However, the intrinsic motivation of nurses to reduce fall incidents is in my opinion determined by communication/marketing.*

*P: Digital communication could have, in my opinion, a major contribution in improving knowledge. Imagining situations by, for instance, Virtual Reality.*

A small number of managers emphasizes more on involving nurses in their communication policy.

*P: In my opinion, to improve the knowledge, attitude and skills of nurses, it is important to have frequent conversations about why, how this can be implemented in daily practice, about nursing leadership and to place responsibility with nurses.*

Guidelines and regulations are often chosen as categories with the means to facilitate nurses.

*P: .It is important that nurses receive the right information, understand the importance, know what is expected of them and receive sufficient support.*

*P: protocols based on guidelines, regulation and legislation should be present. They also should be implemented and be available for nurses.*

#### Opportunity (social and environmental influences)

Most of the managers selected fiscal measures, service provision and environmental / social planning as policy categories to create awareness and stimulate leadership.

*P: the role of the manager is to facilitate nurses who can be initiators. Use the qualities in the team. Facilitate nurses who can use Evidence Based Practice, clinical reasoning and coaching.*

*P: We also create awareness that registrations can lead to awareness in higher management and therefore may lead to action.*

*P: Awareness among managers of misplaced thrift? Integrative approach is necessary in accommodation and logistics structures. Stimulate nursing leadership en facilitate important conditions. Multidisciplinary consultation could help create awareness and improve knowledge.*

#### Motivation (emotions and beliefs)

To influence motivation of nurses managers mainly selected communication/marketing, guidelines, regulation and environmental/social planning as policy strategies. By this they try to influence the intrinsic motivation of nurses, create a cultural change and empower nurses.

*P: nurses need to be empowered. They are trained to analyze, research, improve, etc. To express this in daily practice nurses need coaching, time to think, discuss problems and mental support.*

*P: in my opinion a culture change is necessary for nurses to look at themselves objectively and to be critical of their own work and responsibilities and to look themselves for solutions of nursing problems.*

*P: The intrinsic motivation of nurses is, in my opinion, determined by policy strategies such as communication/marketing, context/social adaptions and possibilities for specific service provision.*

#### Stage three; identify content and implementation options (steps seven and eight)

Steps seven and eight were included in one Delphi round with educational experts. Of the 14 included experts, four educational experts did not complete this round due to time limitations. Educational experts received the results of Table 3 and were asked to give qualitative feedback about the content and way of delivery of the selected educational categories. Results are described below per COM-B component.

#### Capability (knowledge and skills)

Educational experts selected education, incentivisation and modelling as interventions to influence capability. Educational experts mention a diversity of possible examples. Interventions can target asking critical questions about guidelines or discussing a patient case. Nurses should also be stimulated to gain insight in the benefits of fall prevention, such as improving quality of care.

*P: The best method is for nurses to intrinsically realize that it is of upmost importance to work on being capable.*

*P: Nurses should discuss patient cases together. Together with colleagues discuss what new insights were discovered.*

*P: When it comes to knowledge transfer, fall prevention should be priority at an educational moment at the ward. Each week a critical question could be asked about, for instance, a local guideline of patient case.*

#### Opportunity (social, environmental influences)

According to educational experts, nurses should be encouraged to change the physical context they are working in. However, important preconditions such as financial possibilities are necessary.

*P: It is important that knowledge transfer is wide and context based implemented.*

*P: Listening to healthcare professionals is important. How is it possible that materials are out of stock, joint agreements need to be made about that, or checked who is involved and how processes run. Then reward when improvements are found and time needs to be available for implementation.*

Educational experts find modelling the most important intervention to influence social opportunity. Working multidisciplinary together with other nurses, physiotherapists, patients and families is a central theme that can be stimulated by offering opportunities for consultation. The content can aim at, for instance, intervision, patient case reviewing, education and modelling. It is important that nurses have the opportunity to learn with en from each other on the ward.

*P: Multidisciplinary collaboration is increasing in importance. A community of practice can stimulate this. Asking a question at a colleague at the coffee machine is also learning. Shadowing other disciplines gives insight in other functions and processes.*

#### Motivation (emotions, beliefs)

By incentives, environmental restructuring and modelling nurses can be motivated to use and implement fall prevention interventions. By visualization (smart reminders, posters) nurses can be alerted and complimented. Nurses, who are role models, can support colleagues by motivating them. It is also possible to include a competition element to influence motivation.

*P: Why are computer games so popular? Because good games consist of nothing else than a combination of fun and rewards: you want to achieve the next level, you hear positive tunes when you win something, there are extra bonuses and credits to achieve. They are drives to continue. You want to improve yourself or win from your opponent. This competition element can work very powerful.*

#### Link between policy categories and intervention functions

The BCW provides an overview of the link between intervention functions and policy categories. The top three of intervention functions selected by the educational experts are; Incentivisation, modelling and enablement. The top three of policy categories selected by the managers are communication/marketing, regulation and environmental/social planning. When both are integrated in the matrix of Table5 a discrepancy becomes visible. The policy category communication/marketing does not enable nurses, regulation does not support modelling and environmental/ social planning does not incentive nurses and support modelling. A mismatch becomes visible in four out of nine of the selected categories and intervention functions.

**Table 5:**
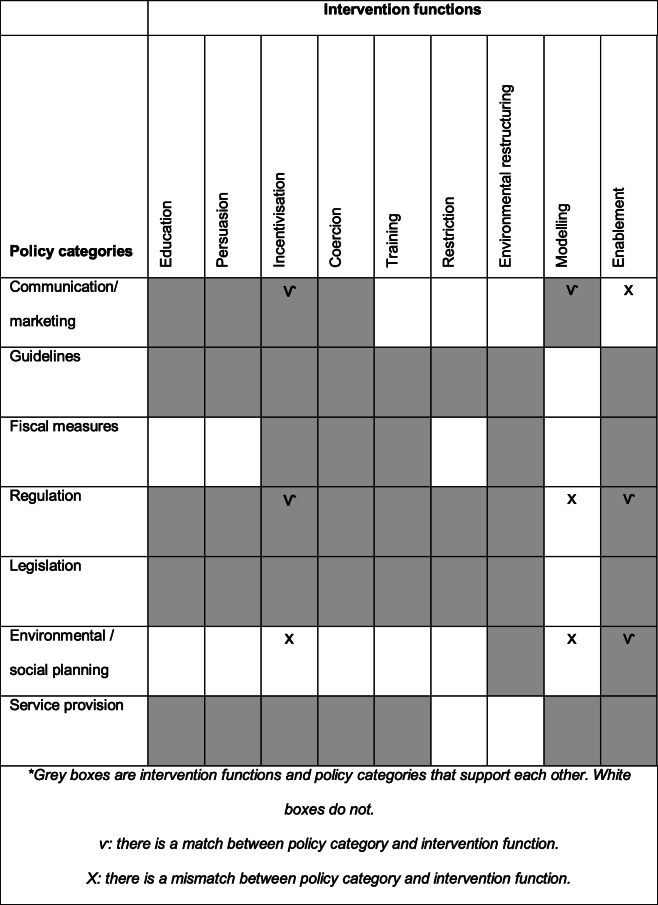
Matrix of links between intervention functions and policy categories^a^

^a^Grey boxes are intervention functions and policy categories that support each other. White boxes do not

: there is a match between policy category and intervention function

X: there is a mismatch between policy category and intervention function

## Discussion

This study aimed to identify interventions to change the behavior of hospital nurses regarding fall prevention among older hospitalized patients. Although several interventions have been identified by different experts, the results especially show the discrepancies between the opinions of nurses, geriatric and educational experts and managers.

The most notable discrepancy became visible between the geriatric experts and hospital nurses. In the experts opinions after-care, estimating fall risk and providing information are the most promising target behaviors (Table 2). However, in nurses opinions the target behaviors should be; providing information, fall prevention and multifactorial fall risk assessment, demonstrating a mismatch in opinions which behaviors should be targeted in a fall prevention program. An important inhibitor in nursing education is the fact that needs of nurses are often not included in the development of educational activities [4, 6]. Those educational activities are often didactic in nature, instead of encouraging nurses to take initiative and direct their own learning [4]. Furthermore, a recent study of Smit et al. showed that education often receives little attention in the development of multicomponent interventions [27]. Thus, to make continuing education programs more effective, nurses need to have a more participatory role in their learning [4]. And the needs of nurses needs to be included in the development of educational programs. The results also underline the need for more collaboration between managers, educational experts and nurses in the development of education programs.

The fact that current education regarding fall prevention may be insufficient may explain why nurses in this study not only report knowledge deficits of others, but also themselves have a lack of knowledge. The lack of knowledge regarding local and national guidelines and the availability of patient information is alarming. Nursing care increasingly involves older patients and nurses knowledge is associated with the quality of care received by older patients [28,30]. As mentioned before, current learning methods are often didactic. Didactic, formal learning interventions lead to few results in workplace and emphasize on education rather than improving ones practice [31]. And therefore, may not change the behavior of nurses and impact patient outcomes [31]. This may explain why the compliance of nurses in this study in applying fall risk assessment and fall prevention is low.

Another notable discrepancy was found between the managers and the educational experts. The selected interventions of the managers and educators show that communication/marketing does not enable nurses in their behavior, regulation does not support modelling and environmental/ social planning does not incentive nurses nor supports modelling (Tables3, 4, 5). There is little research about the important role managers and educators have in empowering nurses to deliver excellent care [32]. A strong collaboration between practice and education, a positive organizational culture and managers supporting role influence the impact of education on practice [33] Managers should create positive cultures by being role models, support personal development plans and by working together with education colleagues [33]. Results also show that collaboration between practice and education providers is difficult [33]. This study demonstrated a mismatch too, and as previous research stated, the facilitation of workplaces that enable excellence in nursing care will not occur without active and improved collaboration between managers and educators [32].

The lack of important preconditions, such as the availability of mobilization tools, also underline the crucial role of workplaces in providing the right care. A result that is consistent with previous research about experienced barriers by nurses for successful fall prevention programs [34]. Nurses take limited control to change these conditions, but when they do, their efforts lead to few results because of managerial decision processes, which influences their motivation in a negative way. Therefore, managers need to facilitate the opportunity for nurses to deliver patient-centered care [21]. A study of Fonda et al. stated that *strong leadership and environmental support were successful strategies for implementing fall prevention programs* [35].

This study included several strategies to maximize trustworthiness. However, there were also some limitations. Often, Delphi studies consist of more rounds than two. Due to time limitations it was not possible to include more rounds. This limited the possibility for more in-depth questions. However, consensus was reached within the different rounds among experts. Due to work load on wards some nurses were not able to attend the focus groups and five hospitals did not succeed in organizing a focus group, this limits the generalizability of the results. However, saturation was reached and the diversity in context led to rich and interesting data. Nine out of ten hospitals participated in the study by including experts and nurses in at least one of the eight steps of the BCW. In this study the BCW was used as a framework to identify interventions for behavior change. The BCW may not have been the best framework to identify those interventions. But following these steps also showed the discrepancies between managers, educational experts and nurses. They were included in the steps of the BCW where their responsibilities in real practice also lies. The results therefore show an important collaboration inhibitor for continuing nursing education in nursing practice.

## Conclusion

Several interventions were identified with the aim to change the behavior of nurses [16]. However, this study specifically showed the mismatch in perceptions between hospital nurses, geriatric experts, educators and managers, regarding how this behavior should be changed. This mismatch should be investigated further, as excellence in nursing care cannot be achieved without active and improved collaboration between nurses, managers and educators. This is also underlined by the knowledge-to-action theories. *Knowledge-to-action is about an exchange of knowledge between relevant stakeholders that results in action. To achieve this, appropriate relationships must be cultivated. This requires the identification of relevant stakeholders and establish a common understanding of knowledge-to-action* [3]. Therefore, to minimize the knowledge-to-action gap, further insight in the role and collaboration of managers, educators and nurses is necessary for the development of education programs strengthening change at the workplace and enable excellence in nursing practice.

## Supplementary Information

**Additional file 1.** Interview guide focus groups with nurses.

**Additional file 2.** Step 2 Behavior Change Wheel.

**Additional file 3.** Diagram of data focus groups: Capability.

**Additional file 4.** Diagram of data focus groups: Opportunity.

**Additional file 5.** Diagram of data focus groups: Motivation.

## Data Availability

The datasets used and/or analyzed during the current study are available from the corresponding author on reasonable request.

## Abbreviations

BCW: Behavior Change Wheel
COM-B: Capability, Opportunity, Motivation Behavior
RENursE: Research, Education, Nursing regarding Elderly
I-CVI: Item content validation index
NR: Nurse researcher
