# Supplementary material for: Minimizing the knowledge-to-action gap; identification of interventions to change nurses' behavior regarding fall prevention, a mixed method study
Source: BMC Nurs. 2021 May 21;20:80. doi: 10.1186/s12912-021-00598-z (PMC8139083; doi:10.1186/s12912-021-00598-z)
Supplement: Supplementary file 1 — Additional file 1. Interview guide focus groups with nurses. [file 12912_2021_598_MOESM1_ESM.docx]

**Additional file 1, Interview guide focus groups with nurses.**

| Phase 0:  General opening | What comes to your mind when you think of fall prevention?  Do you know any protocol or guideline regarding fall prevention?  Are there information leaflets available for patients and family members? |
| --- | --- |
| Phase 1:  Presentation of case study | Presentation of case study involving 70 year older patient with high risk of fall incident.  Questions relating to case study:  What happens if patient in case study presents himself at your ward? |
| Phase 2:  Facilitators  Barriers | What are facilitators for applying fall prevention interventions?  What are barriers for applying fall prevention interventions? |
| Phase 3:  Facilitators and barriers relating to COM-B model. | Capability (Knowledge and skills)  Are you capable in applying fall prevention?  Opportunity (Social, Physical)  Do you have the opportunity to apply fall prevention?  Motivation (automatic, reflective)  Are you motivated to apply fall prevention? |
