# Supplementary material for: Minimizing the knowledge-to-action gap; identification of interventions to change nurses' behavior regarding fall prevention, a mixed method study
Source: BMC Nurs. 2021 May 21;20:80. doi: 10.1186/s12912-021-00598-z (PMC8139083; doi:10.1186/s12912-021-00598-z)
Supplement: Supplementary file 2 — Additional file 2. Step 2 Behavior Change Wheel. [file 12912_2021_598_MOESM2_ESM.docx]

**Additional file 2, Step 2 Behavior Change Wheel**

| **Target behaviors** | **Impact of behavior change** | **Likelihood of changing behavior** | **Spillover score** | **Measurement score** |
| --- | --- | --- | --- | --- |
| Risk assessment |  |  |  |  |
| Multifactorial fall risk assessment |  |  |  |  |
| Fall prevention interventions |  |  |  |  |
| Compliance of elderly to fall prevention plan |  |  |  |  |
| Organization of care |  |  |  |  |
| Education and communication |  |  |  |  |
| Aftercare (after fall incident) |  |  |  |  |
| Rate as: unacceptable, unpromising but worth considering, promising, very promising | | | | |
