# Supplementary material for: Minimizing the knowledge-to-action gap; identification of interventions to change nurses' behavior regarding fall prevention, a mixed method study
Source: BMC Nurs. 2021 May 21;20:80. doi: 10.1186/s12912-021-00598-z (PMC8139083; doi:10.1186/s12912-021-00598-z)
Supplement: Supplementary file 4 — Additional file 4. Diagram of data focus groups: Opportunity. [file 12912_2021_598_MOESM4_ESM.docx]

**Additional file 4; Diagram of data focus groups: Opportunity.**
